# Impact of Isotonic Beverage on the Hydration Status of Healthy Chinese Adults in Air-Conditioned Environment

**DOI:** 10.3390/nu9030242

**Published:** 2017-03-07

**Authors:** Phei Ching Siow, Wei Shuan Kimberly Tan, Christiani Jeyakumar Henry

**Affiliations:** 1 Clinical Nutrition Research Centre (CNRC), Singapore Institute for Clinical Sciences (SICS), Agency for Science, Technology and Research (A*STAR) and National University Health System, Centre for Translational Medicine, 14 Medical Drive #07-02, MD 6 Building, Yong Loo Lin School of Medicine, Singapore 117599, Singapore; siow_phei_ching@sics.a-star.edu.sg (P.C.S.); Kimberly_tan@sics.a-star.edu.sg (W.S.K.T.); 2 Department of Biochemistry, National University of Singapore, 8 Medical Drive, Singapore 117596, Singapore

**Keywords:** hydration, air-conditioning, indoor, isotonic beverage, fluid retention

## Abstract

People living in tropical climates spend much of their time in confined air-conditioned spaces, performing normal daily activities. This study investigated the effect of distilled water (W) or isotonic beverage (IB) on the hydration status in subjects living under these conditions. In a randomized crossover design, forty-nine healthy male subjects either consumed beverage or IB over a period of 8 h (8 h) in a controlled air-conditioned environment. Blood, urine, and saliva samples were collected at baseline and after 8 h. Hydration status was assessed by body mass, urine output, blood and plasma volume, fluid retention, osmolality, electrolyte concentration and salivary flow rate. In the IB group, urine output (1862 ± 86 mL vs. 2104 ± 98 mL) was significantly lower and more fluids were retained (17% ± 3% vs. 7% ± 3%) as compared to W (*p* < 0.05) after 8 h. IB also resulted in body mass gain (0.14 ± 0.06 kg), while W led to body mass loss (−0.04 ± 0.05 kg) (*p* = 0.01). A significantly smaller drop in blood volume and lower free water clearance was observed in IB (−1.18% ± 0.43%; 0.55 ± 0.26 mL/min) compared to W (−2.11% ± 0.41%; 1.35 ± 0.24 mL/min) (*p* < 0.05). IB increased salivary flow rate (0.54 ± 0.05 g/min 0.62 ± 0.04 g/min). In indoor environments, performing routine activities and even without excessive sweating, isotonic beverages may be more effective at retaining fluids and maintaining hydration status by up to 10% compared to distilled water.

## 1. Introduction

In a warm, humid, tropical climate like Singapore, air-conditioning is provided in workplaces and classrooms with the view that it will enhance workplace productivity and thermal comfort [1]. People spend 80%–90% of their time indoors [2]. Lower air temperature and relative humidity of an air-conditioned room increase water losses via skin. Moreover, air velocity and choice of clothing also affect the rate of water loss. The water loss via skin in healthy adults may be up to 0.3 L/h and via urine around 1–2 L per day [3]. 

Water constitutes 60% of the body weight of healthy adults. It is often overlooked as an essential nutrient that affects the body’s performance both mentally and physically [4,5]. Proper hydration aids in the elimination of toxins, efficiency in digestion, joint lubrication, thermoregulation, and other biochemical reactions [6]. The human body constantly loses water through the respiratory system, urinary system, respiratory tract, and gastrointestinal system—even without visibly sweating. The body can gain water through food and drink as well as metabolic production [7,8]. It has been extensively studied that strenuous labour, exercise, and heat stress can induce excessive water loss. Nevertheless, hydration status of people living in a mild environment has not been studied often.

Many indices have been developed in the past 20 years to accurately assess hydration status in humans [9]. These include both invasive and non-invasive methods. Plasma osmolality, urine osmolality, specific gravity, and colour are the most widely used markers. Since the extracellular fluids move into and out of the circulation, it stimulates important fluid-regulatory mechanisms. Thus, change in body mass, haematological and urine parameters, and bioelectrical impedance are important indicators to evaluate the extracellular water changes before and after treatments. Heart rate and blood pressure are the other common hydration status markers [8,9,10]. 

The composition of fluid ingested is as essential as the volume. Isotonic beverages consisting of water, carbohydrate, and electrolytes are solutions that contain the same osmolality as blood, where the osmolality is normally regulated between 280 and 290 mOsm/kg by controlling water loss and intake [11,12]. It aims to restore fluid and electrolyte homeostasis. It is often consumed during sports events where a large amount of water and electrolytes are lost through sweat. Previous studies demonstrated that the inclusion of sodium and carbohydrate in a beverage enhanced fluid retention and restored fluid loss after thermal [13], exercise [13,14,15,16,17,18], or fluid restriction [19,20] induced dehydration. However, the effect of such drinks in an air-conditioned environment without excessive dehydration has not been extensively studied. In order to test the effect of carbohydrate and electrolytes in combination (as found in isotonic beverages), we used distilled water as the non-electrolyte control. The decision to use distilled water was based on the fact that consumers in Singapore drink both bottled and tap water of varying mineral content. 

Although hydration strategies during exercise and are fundamental, hydration in air-conditioned environments is also important for maintaining optimum working performance. In most studies, dehydration was induced by water restriction, exposure to heat stress, or physical exercise, and rehydration was achieved by providing plain water. In our study, the measurement of hydration status was used to provide new insight into the physiological status in an indoor air-conditioned environment. 

## 2. Materials and Methods

### 2.1. Participants

Fifty-two healthy Chinese male subjects aged 21–50 years with a BMI between 19 and 26 kg/m^2^ and fasting blood glucose <5.5 mmol/L were recruited. Subjects with food allergy, disliking the consumption of isotonic beverage, smoking, on prescription medication or partaking in competitive sports were excluded. The study had prior approval from the local Institutional Review Board (reference no. DSRB 2015/00322). The protocol was well explained to the subjects, and written informed consent was obtained. The screening process was conducted to make sure the subjects met the inclusion criteria. Forty-nine subjects completed the study. One subject dropped out from the study due to work commitments and two due to difficulty in drawing venous blood. The baseline characteristics of the subjects are provided in Table 1.

### 2.2. Pre-Trial Standardisation

Preceding each test day, strenuous exercise and consumption of alcohol, caffeine, supplements, and medications were prohibited. Subjects were provided with a standardized dinner on the day before the test session. Bottled distilled water was also given to the subjects to consume with the dinner. Subjects were instructed to consume the water ad-libitum up to 30 min before the test commenced the next morning. On the first test session, the bottled water was returned and the volume of water consumed was recorded. To standardize the amount of water consumed before the experiment, subjects drank the same amount of water before the next test session. 

### 2.3. Study Design

The study was a randomized cross-over design. Subjects completed two test sessions on non-consecutive days, consuming either distilled water (W) or isotonic beverage (IB). The order of treatments was randomized using “Research Randomizer” [21]. During the study, standardized breakfast, lunch, and afternoon snack were provided to the subjects. Equal volume (325 mL) of IB or W was consumed with each of the standardized meals. Subjects were required to stay in an air-conditioned room (24.43 ± 0.07 °C; 55.51% ± 0.33% RH) throughout the 8 h. 

On the test day, subjects reported to the laboratory in the morning after an overnight fast. Subjects were instructed to void their bladder, collecting the entire volume. Subjects’ body mass was then measured using an electronic weighing and measuring station (SECA 763, Hamburg, Germany). Hip and waist circumferences were measured using a measuring tape. Blood pressure and heart rate were measured using digital blood pressure monitor (OMRON HEM-907, Kyoto, Japan). Total body water (TBW) was measured using bioelectrical impedance analyser (BIA) (Tanita BC 418, Tokyo, Japan). Baseline blood sample was collected by finger prick and venepuncture. Baseline saliva sample was collected in salivette tube. After measurements and sample collection, subjects were provided with standardised breakfast, lunch, and afternoon snack at time 0, 3 h, and 6 h, respectively. Aside from the drinks provided with the meals, the subject was also given 1500 mL of W or IB during the first session for ad-libitum consumption. More drinks were available if the subjects finished the drinks provided. They were instructed to record the amount and time when they consumed the drink. The total amount consumed was recorded and the subject was asked to duplicate the drinking pattern during the next visit. Therefore, the volumes of beverage or IB consumed were the same in both groups. Subjects also collected all urine produced over the 8-h period and refrained from any physical activity for the duration of each group. Eight hours after breakfast, anthropometric measurements, physiological measurements, and biological samples collection were performed as in the morning. 

### 2.4. Study Meals

Standardised dinner consisted of breaded fish in tamarind sauce with rice (CP Ready Meals, Singapore) and mango pudding (Naspac, Thailand). Standardised breakfast was three slices of white bread (Gardenia, Singapore) and margarine (Naturel Soft, Malaysia). Standardised lunch was teriyaki chicken with rice and glazed teriyaki chicken. Standardised afternoon snack was almonds (Camel, Singapore). The same standardized meal was fully consumed throughout the study. The isotonic beverage used contained both carbohydrate and electrolytes. The composition of the distilled water (Alpheus, Singapore) and isotonic beverage (F & N, Singapore) is presented in Table 2. 

### 2.5. Urine, Blood, and Saliva Biomarkers

Blood sample was drawn from an antecubital vein. Part of the blood samples were collected into BD Vacutainer^®^ Plus Plastic K_2-_EDTA tubes (Becton, Dickinson and Company, Franklin Lakes, NJ, USA) for Full Blood Count test (FBC) which analysed haemoglobin concentration and haematocrit. The remainder of the blood samples was allowed to clot in BD Vacutainer^®^ Serum tubes (Becton, Dickinson and Company, Franklin Lakes, NJ, USA) for 30 min at room temperature before centrifugation (Sorvall™ ST 16 Centrifuge, Thermo Fisher Scientific Inc., Waltham, MA, USA) for 10 min at 4 °C at 1500× *g*. Subjects collected all urine produced during the 8 h duration in a 3-L plastic urine bottle (Simport Scientific, Beloeil, QC, Canada). The urine bottle was then measured on an electronic balance and the mass of the empty urine bottle was subtracted to obtain the total mass of the urine. Saliva samples were collected using pre-weighed salivette tubes (Sarstedt, Nümbrecht, Germany). Subject was asked to swallow in order to standardise the amount of residual saliva prior to collection. The cotton swab was then placed under the tongue for 2 min with minimum orofacial movements during the collection. The salivette tube was then weighed immediately after collection and centrifuged for 2 min at 1500× *g*.

Serum, urine, and saliva samples were analysed for osmolality by freezing point depression (MicroOsmometer 3320, Advanced Instrument, Norwood, MA, USA). Osmolality was measured in duplicate within 3 h of collection. Serum and urine sodium, potassium, and chloride concentrations were also analysed. Haemoglobin and haematocrit values were used to estimate percentage changes in blood and plasma volumes according to the equation proposed by Dill and Costill [22]. Urine specific gravity (USG) was measured using a handheld refractometer (Atago, Tokyo, Japan). The volume was determined by multiplying the urine mass and USG. Urine colour was determined using eight-point scale urine colour chart [23] with a numbered scale ranging from very pale yellow (number 1) to brownish green (number 8). Salivary flow rate was calculated by dividing the difference in the weight of salivette tube before and after collection by collection time. Fluid retention was calculated based on the fluids ingested from drink and foods and urine output using a standard equation [15]. Urine flow rates were also calculated and used to estimate free water clearance ($C_{H_{2}O}$) [18]:

$$C_{H_{2}O}=V\times \left(1-\frac{U_{osm}}{S_{osm}}\right),$$

where *V* is urinary flow rate in 8 h, *U_osm_* is urine osmolality, and *S_osm_* is serum osmolality.

### 2.6. Statistical Analysis

A total of 49 subjects were analysed. Data were analysed using paired *t*-test. Level of significance was taken at *p* < 0.05. Statistical analysis was performed using SPSS software (SPSS Inc., Chicago, IL, USA), version 23.0. Data were presented as mean ± standard error (SE). Due to insufficient saliva and urine volume for analysis in one subject, the data presented were from 48 subjects where we have a complete data set. In addition, full blood count was not performed in three samples, as the blood coagulated before testing; therefore, the blood and plasma volume were calculated based on the results of 46 subjects. 

## 3. Results

The hydration indicators before and after treatments are tabulated in Table 3. A total of 49 subjects completed the study. After 8 h in the air-conditioned environment (24.43 ± 0.07 °C; 55.51% ± 0.33% RH), change in body mass was significantly different between both groups (*p* = 0.01). Isotonic beverage resulted in body mass gain (0.14 ± 0.06 kg), while body mass loss (−0.04 ± 0.05 kg) was observed in the distilled water group. However, the change in body water measured by BIA was not significantly different between two groups (Table 3). Lower urine volume was produced in the isotonic beverage group (1861.65 ± 85.82 mL) compared to the distilled water group (2104.61 ± 97.69 mL) (*p* = 0.001) over the duration of the study (i.e., 8 h). Isotonic beverage consumption retained 17% of fluid, while distilled water consumption retained 7% of fluids (*p* = 0.001) (Table 4). C_H2O_ was lower in the isotonic beverage group than in the distilled water group (*p* = 0.001). Blood volume decreased to a significantly greater extent in the distilled water group compared to the isotonic beverage group (*p* < 0.05). In addition, distilled water consumption resulted in a greater decrease in plasma volume (−3.62% ± 0.83%) compared to isotonic beverage consumption (−1.85% ± 0.81%), although no statistical difference was observed (*p* = 0.056).

Consumption of both distilled water and isotonic beverage in an air-conditioning environment for 8 h led to a reduction in urine osmolality, [Na^+^], [K^+^], and [Cl^−^]. Nevertheless, a significantly lesser drop in urine [Na^+^] and [Cl^−^] was found in the isotonic beverage group compared to the distilled water group (*p* < 0.05), indicating that more Na^+^ and Cl^−^ were excreted through urine in the isotonic group than in the distilled water group. A higher reduction in urine osmolality was observed in the distilled water group than in the isotonic beverage group (−374.43 ± 43.83 mOsm/kgH_2_O vs. −295.84 ± 43.89 mOsm/kgH_2_O) (*p* =0.052). USG decreased from baseline in both groups, with no significant group effect regarding the relative changes. Similarly, urine colour was significantly paler after 8 h in both groups (*p* < 0.05), with no difference between groups (*p* > 0.05).

A significant difference was found in the change in serum [Na^+^] between two groups (*p* = 0.005). Serum [Na^+^] increased significantly after isotonic beverage consumption (139.91 ± 0.28 mmol/L to 140.59 ± 0.24 mmol/L) (*p* < 0.01), while a reduction in serum [Na^+^] was observed after distilled water consumption (140.41 ± 0.30 mmol/L to 139.69 ± 0.33 mmol/L) (*p* = 0.053). Both groups showed a significant increase in serum [K^+^] (*p* < 0.05) after 8 h, with no group effect. No significant difference was found in the changes in serum [Cl^−^]. Lastly, 8 h consumption of isotonic beverage in an air-conditioned room resulted in a significant increase in serum osmolality (290.46 ± 0.71 mOsm/kgH_2_O to 295.10 ± 0.98 mOsm/kgH_2_O), although no significant difference was found between groups.

Salivary flow rate increased from 0.54 ± 0.05 g/min to 0.62 ± 0.04 g/min in the isotonic beverage group (*p* < 0.05), whereas no changes were observed after distilled water consumption. Saliva osmolality decreased in the distilled water group (*p* < 0.05), while remaining unaltered in the isotonic beverage group. Nevertheless, no significant difference was found between groups for both saliva flow rate and osmolality. Although no difference was found between groups, systolic blood pressure significantly increased from 114.96 ± 1.49 mmHg to 117.53 ± 1.46 mmHg after isotonic beverage ingestion (*p* < 0.05), whereas no difference was found in distilled water group (from 117.43 ± 1.54 mmHg to 117.82 ± 1.68 mmHg). Heart rate was found to be decreased significantly (*p* < 0.01) in both groups. 

## 4. Discussion

As far as we are aware, this is the first study to demonstrate that in a confined indoor air-conditioned environment, the use of isotonic beverage has a beneficial impact on hydration. Much research has been conducted in extreme tropical environments, with limited research conducted under indoor conditions. The present study suggests that the ingestion of isotonic beverage may be more effective in retaining fluid even in indoor environment—notably, an air-conditioned space. These observations are important, as people today lead busy lives confined to indoor spaces for most of the day.

Our results showed that fluid retention was 10% higher with isotonic beverage compared to distilled water (IB 17%, W 7%). The cumulative urine output was 240 mL less with isotonic beverage than with distilled water, and the free water clearance was lower after isotonic beverage consumption. In addition, there was a smaller drop in plasma volume and blood volume after isotonic beverage ingestion. Subjects also experienced body mass gain in the isotonic beverage group (due to greater water retention) as compared to the distilled water group, which resulted in body mass reduction. In addition, serum and urine sodium concentrations reflected the sodium content in the drinks administered. 

The isotonic beverage used in this study contained 6% carbohydrate, 21 mmol/L sodium, 4 mmol/L potassium, and 11 mmol/L chloride. The presence of these carbohydrates and electrolytes played an important role in maintaining hydration status. Studies have shown that electrolytes—particularly sodium—enhance fluid retention in the extracellular space, restore fluid homeostasis, and maintain plasma concentrations of vasopressin and aldosterone [13,18,24]. As a result, it prevented diuresis and maintained plasma and blood volume. This explains the lower urine output observed in this study. In addition, glucose in the isotonic beverage also stimulated glucose and solute absorption in the small intestine, thus increasing fluid absorption [13,25]. It has been reported that the absorption of glucose at the jejunum is coupled with two sodium and 300 water molecules [26]. Therefore, more fluids can be absorbed in the isotonic beverage group. Water—which does not contain any carbohydrate—limited the total solute absorption, and hence fluid delivery. 

Furthermore, the carbohydrate and electrolyte contents in the isotonic beverage contribute to the higher beverage osmolality. Higher osmolality will then stimulate more transport mechanisms, leading to greater water absorption [27]. In addition, it has been reported that the fluid-regulating hormone, arginine vasopressin (AVP) is sensitive to slight increases in plasma osmolality, and it fluctuates by 0.41 pmol/L per unit change in plasma osmolality. Therefore, it is likely that AVP was stimulated by the elevation in serum osmolality in the isotonic beverage group, promoting the reabsorption of water by the kidney and resulting in better fluid retention [18,28,29]. Furthermore, C_H2O_—a good estimation of AVP responses—was lower with the isotonic beverage, resulting in lower urine output. 

There was a larger amount of sodium and chloride excreted in the urine after drinking isotonic beverage than drinking distilled water, with no difference in the total amount of potassium excreted. This may be due to the contribution of sodium and chloride in the isotonic beverage. In addition, this higher electrolyte excretion may also explain the higher urine osmolality and urine specific gravity after consumption of the isotonic beverage. 

Each subject consumed the same amount of foods and drinks, and the environment was consistent across the two groups; therefore, changes in body mass may be taken as a valid indicator of hydration status in both groups. Acute changes in body mass over a short time period can be assumed to be due to body water loss or gain, as no other body components can be lost at such a rate [7,8]. Therefore, it can be said that more water was retained after isotonic beverage consumption. Nevertheless, the TBW measured by BIAs did not show significant difference between distilled water and isotonic beverage. This may be because BIA is a poor instrument to measure TBW and extracellular water (ECW) [30,31]. 

A urine specific gravity of ≤1.020, urine osmolality of ≤700 mOsm/kgH_2_O, urine volume of approximately 100 mL/h, and body weight change of <1% are the indicators of euhydration [9,32,33]. The results after 8-h consumption of both distilled water and isotonic beverage showed that the subjects were all well hydrated. Subjects were required to fully consume a fixed amount of beverages with meals and were provided with additional volumes of beverage for ad-libitum consumption. This suggests that continuous consumption of fluids is effective to achieve euhydration. 

Salivary flow also increased by 15% from baseline after consumption of isotonic beverage. This might be due to the presence of salt and sugar in the isotonic beverage, as saliva secretion can be stimulated by taste [34]. The increase in salivary secretion may have some importance in people who often suffer from dry mouth and reduced salivary flow. This may be of special relevance in the elderly, who show decreased salivary flow. The presence of saliva in the oral cavity stimulates gustatory receptors on the taste buds, and is essential for taste perception. Food particles need to be in solution in order to stimulate taste receptor cells in the taste buds within the lingual papillae. Furthermore, saliva facilitates speech, mastication, and swallowing [34,35]. This observation necessitates further research to confirm the utility of isotonic beverages to facilitate salivary flow and enhance gustatory stimulation in the elderly.

A statistically significant increase in blood pressure and decrease in heart rate was found in the isotonic beverage group, but such difference might not be physiologically significant. Blood volume, blood pressure, and heart rate are closely linked. Water intake can acutely reduce heart rate and increase blood pressure in normotensive individuals, and this effect can last for up to 60 min [5,36]. Furthermore, Jormeus reported that the changes in blood pressure were the consequences of the amount of water per se, and not due to the potential confounders such as changes in salt intake [37]. Therefore, the increase in blood pressure in the isotonic beverage group was likely due to the better water retention and hydration status instead of the salt intake, and the slight changes in heart rate are to balance the effect of normal fluctuations in blood volume on blood pressure [5]. 

There are some limitations in this study. Further research should be performed to evaluate the rate of water loss in subjects living in air-conditioned environments. Secondly, 24 h urine needs to be collected to evaluate the hydration status of the subjects throughout the entire day. Since this study was conducted under constant temperature and humidity, it is impossible to extrapolate what the results will be when the subjects are confined in varying temperature and humidity. Although our results demonstrate the advantage of consuming isotonic beverage in certain circumstances, it does not invalidate the possibility that hypertonic beverages may also have similar effects. The independent effect that sucrose or electrolytes may have on hydration status separately needs to be further investigated. Finally, the consumption of isotonic beverage through the entire day may increase individuals’ sugar intake and desire for consuming sweet beverages. 

## 5. Conclusions

In conclusion, in an indoor air-conditioned environment with minimal physical activity, the consumption of isotonic beverage promoted fluid retention more than distilled water. Given the large number of people working and residing indoors under air conditioning throughout the day, these results suggest that the consumption of isotonic beverages may be more beneficial than water in order to stay hydrated and to maintain fluid balance. Though not the primary intention of our study, the novel observation that salivary secretion was enhanced in those consuming isotonic beverages indicates that especially in the elderly, the use of isotonic beverages may promote greater appreciation of food due to the role salivary secretion plays in taste perception. 

## Figures and Tables

**Table 1:** Baseline characteristic of subjects.

Anthropometric and Physiological Parameters	*n* = 49
Age	25 ± 0.75
Weight, kg	67.8 ± 1.01
Height, cm	175.0 ± 0.86
BMI, kg/m^2^	22.1 ± 0.22
Waist circumference, cm	74.6 ± 0.66
Hip circumference, cm	95.0 ± 0.55
Total body water, kg	37.8 ± 0.55
Systolic blood pressure, mmHg	117 ± 1.54
Diastolic blood pressure, mmHg	70 ± 1.17
Heart rate, beats/min	65 ± 1.27
Blood glucose, mmol/L	4.6 ± 0.06

All the data is presented as mean ± SE.

**Table 2:** Composition of distilled water and isotonic beverage.

Nutrient Values	Distilled Water	Isotonic Beverage
Osmolality ^1^, mOsm/kg H_2_O	0	284
Sodium concentration ^2^, mmol/L	0	20.87
Potassium concentration ^2^, mmol/L	0	3.59
Chloride concentration ^2^, mmol/L	0	10.99
Carbohydrates ^2^, g/L	0	60
Simple sugars ^2^, g/L	0	60
Fat ^2^, g/L	0	0
Protein ^2^, g/L	0	0

^1^ Measured by freezing point depression; ^2^ Calculated from product nutrition information panel (NIP).

**Table 3:** Hydration indicators before and after 8 h consumption of distilled water or isotonic beverage.

Biological Parameters	Distilled Water		Isotonic Beverage		*p* (Change between Groups)
	0 h	8 h	0 h	8 h	
Body mass, kg	67.76 ± 1.02	67.72 ± 1.01	67.61 ± 1.05	67.74 ± 1.04 ^†^	0.01
Body water, kg	37.78 ± 0.55	36.62 ± 0.50 ^†^	37.68 ± 0.56	36.66 ± 0.53 ^†^	0.316
Systolic blood pressure, mmHg	117.43 ± 1.54	117.82 ± 1.68	114.96 ± 1.49	117.53 ± 1.46 ^†^	0.139
Heart rate, beats/min	65.12 ± 1.27	60.9 ± 1.44 ^†^	64.12 ± 1.39	61.51 ± 1.20 ^†^	0.259
Blood glucose, mmol/L	4.62 ± 0.01	4.6 ± 0.01	4.72 ± 0.01	4.71 ± 0.01	0.882
Urine specific gravity (USG)	1.0168 ± 0.0010	1.005 ± 0.0005 ^†^	1.0166 ± 0.0072	1.0067 ± 0.0006 ^†^	0.069
Urine colour (scale 1–8)	4.51 ± 0.27	1.46 ± 0.13 ^†^	4.58 ± 0.33	1.66 ± 0.1 ^†^	0.663
Urine osmolality, mOsm/kg H_2_O	600.82 ± 41.34	226.4 ± 17.19 ^†^	573.08 ± 42.02	277.24 ± 21.84 ^†^	0.052
Urine [Na^+^], mmol/L	75.56 ± 6.97	49.02 ± 3.21 ^†^	68.67 ± 6.14	64.85 ± 5.06	0.002 *
Urine [K^+^], mmol/L	42.85 ± 3.79	16.21 ± 1.45 ^†^	41.60 ± 3.71	17.85 ± 1.58 ^†^	0.483
Urine [Cl^−^], mmol/L	83.21 ± 7.62	44.89 ± 2.98 ^†^	78.15 ± 7.03	58.46 ± 4.23 ^†^	0.005 *
Serum osmolality	291.59 ± 0.77	293.9 ± 1.33	290.46 ± 0.71	295.1 ± 0.98 ^†^	0.253
Serum [Na^+^], mmol/L	140.41 ± 0.3	139.69 ± 0.33	139.91 ± 0.28	140.59 ± 0.24 ^†^	0.005 *
Serum [K^+^], mmol/L	4.36 ± 0.05	4.50 ± 0.05 ^†^	4.35 ± 0.05	4.54 ± 0.05 ^†^	0.571
Serum [Cl^−^], mmol/L	102.59 ± 0.26	102.18 ± 0.23	102.51 ± 0.21	102.39 ± 0.2	0.35
Salivary flow rate, g/min	0.65 ± 0.05	0.66 ± 0.04	0.54 ± 0.05	0.62 ± 0.04 ^†^	0.153
Saliva osmolality, mOsm/kg H_2_O	63.27 ± 2.37	57.8 ± 1.64 ^†^	60.76 ± 2.04	60.46 ± 1.65	0.089

Data are presented as mean ± SE. ^†^ denotes significant difference from baseline to after 8 h of drink consumption (*p* < 0.05). * denotes significant difference between groups (*p* < 0.05).

**Table 4:** Effects of distilled water and isotonic beverage on urine volume and blood volume (BV) and plasma volume (PV) changes.

Fluid intake and Hydration Indicators	Water	Isotonic Beverage	*p*
Drink volume, mL	1934.90 ± 66.70	1941.22 ± 65.94	0.160
Water content in food, mL	300.02 ± 0.99	300.02 ± 0.99	
Total urine output, mL	2104.61 ± 97.69	1861.65 ± 85.82	0.001 *
Fraction retained,%	6.98 ± 3.09	17.45 ± 3.02	0.001 *
Free water clearance (C_H2O_), mL/min	1.35 ± 0.24	0.55 ± 0.26	0.001 *
Change in BV, %	−2.11 ± 0.41	−1.18 ± 0.43	0.034 *
Change in PV, %	−3.62 ± 0.83	−1.85 ± 0.81	0.056

Data are presented as mean ± SE. * denotes significant difference between groups (*p* < 0.05).
